# Effects of standardized parenteral nutrition on electrolyte balance: an observational study in preterm neonates

**DOI:** 10.3389/fnut.2026.1849396

**Published:** 2026-10-05

**Authors:** Maria Di Chiara, Francesca Lucci, Arianna Di Domenico, Fabiana Russo, Lucia Dito, Paola Favata, Gianluigi Laccetta, Gianluca Terrin

**Affiliations:** Department of Maternal and Child Health, Policlinico Umberto I, Sapienza University, Rome, Italy

**Keywords:** electrolyte imbalance, individualized parenteral nutrition, NICU, Numeta, parenteral nutrition, preterm neonate, standardized parenteral nutrition, VLBW

## Abstract

**Background:**

Preterm neonates require parenteral nutrition (PN) during the first days of life due to gastrointestinal immaturity. Two main formulations are available: individualized parenteral nutrition (IPN) and standardized parenteral nutrition (SPN). The literature remains inconclusive regarding their comparative safety, particularly concerning electrolyte balance. The aim of this cohort study was to evaluate whether an association exists between different PN formulations and the development of electrolyte disturbances in preterm infants.

**Methods:**

This single-center retrospective observational cohort study included all neonates with gestational age ≤34 weeks and/or birth weight ≤1,500 g admitted to the NICU of Policlinico Umberto I, Sapienza University of Rome, who received PN for at least 3 days between January 2015 and December 2024. Cohort A (*n* = 362) received IPN and Cohort B (*n* = 33) received SPN (Numeta^®^ G13%E). Electrolyte disturbances, nutritional intakes, and morbidities were compared. Binary logistic regression was used to identify independent risk factors.

**Results:**

No significant differences in baseline clinical characteristics were observed between groups. Use of SPN was associated with significantly lower intakes of sodium and calcium during the first week of life. In multivariable analysis, SPN was independently associated with hyponatremia (OR 38.9, *p* < 0.001), hyperkalemia (OR 2.5, *p* = 0.021), hypocalcemia (OR 4.1, *p* < 0.001), and hyperphosphatemia (OR 7.1, *p* < 0.001).

**Conclusion:**

In this study, standardized parenteral nutrition was associated with a significantly higher risk of electrolyte and mineral imbalances compared with individualized formulations. Where SPN is used, supplementation strategies should be considered to prevent complications.

## 1 Introduction

Preterm newborns are at increased risk of morbidity and mortality, and their nutritional requirements are particularly high (1–3). During the first days of life, enteral nutrition (EN) alone is often insufficient to meet these needs because of gastrointestinal immaturity. In this context, parenteral nutrition (PN) plays a crucial role in ensuring survival and supporting postnatal growth. Indeed, early nutritional intake has been shown to influence not only somatic growth but also neurodevelopment and gastrointestinal maturation in preterm neonates (4–7). Nutritional requirements are not constant over time and are particularly demanding during the first week of life, a critical metabolic window compared with subsequent, more stable phases.

Currently, two main PN formulations are available for neonatal care: individualized parenteral nutrition (IPN) and standardized parenteral nutrition (SPN) (8). Both approaches have specific advantages and limitations that should be carefully considered in clinical practice.

Individualized formulations are prescribed daily by the attending physician according to each patient’s individual clinical condition and are compounded by hospital pharmacies or external compounding centers. Their main advantage lies in their high degree of personalization, allowing precise adjustment to the metabolic and clinical needs of each neonate. However, this strategy requires highly trained staff and is associated with higher costs compared with standardized solutions (9, 10).

In contrast, SPN formulations are easier to store and administer, chemically stable, and immediately available. They are also associated with lower costs and a reduced risk of contamination. These formulations do not require daily prescription changes or the continuous availability of hospital pharmacy services (10).

Despite these advantages, SPN also presents relevant limitations. Standardized formulations may not be adequately tailored to the broad spectrum of clinical conditions encountered in preterm neonates, particularly in cases of extreme prematurity, severe illness, or rapidly changing metabolic requirements during the early postnatal period.

The current literature remains inconclusive regarding the superiority of one formulation over the other in terms of efficacy and safety. Most published studies comparing SPN and IPN have focused on macronutrient intake and growth outcomes, whereas electrolyte and mineral balance has received considerably less attention (11). Previous studies generally did not distinguish between the early critical phase and the subsequent period of relative clinical stability. No study has been specifically designed to investigate electrolyte and mineral disturbances as the primary endpoint in a direct comparison between SPN and IPN in preterm neonates, nor to identify independent risk factors for each specific electrolyte abnormality.

Therefore, the aim of the present study was to evaluate whether an association exists between different parenteral nutrition formulations and the development of electrolyte disturbances in preterm infants during the early critical window of the first week of life.

## 2 Materials and methods

### 2.1 Ethics approval and informed consent

The study was conducted in accordance with the World Medical Association Declaration of Helsinki and was approved by the Ethics Committee of Policlinico Umberto I, Sapienza University of Rome (approval no. 5089). Written informed consent was obtained from the parents or legal guardians of all enrolled neonates.

### 2.2 Study population

We included all newborns with a gestational age (GA) ≤ 34 weeks and/or a birth weight (BW) ≤ 1,500 g consecutively admitted to the Neonatal Intensive Care Unit (NICU) of Policlinico Umberto I, Sapienza University of Rome, who received PN for at least 3 days between January 2015 and December 2024. Inclusion criteria additionally required survival beyond the first 24 h of life and absence of major congenital malformations, inborn errors of metabolism or congenital infections. Major congenital malformations included: (i) major cardiac malformations (excluding isolated patent ductus arteriosus); (ii) gastrointestinal disorders, including congenital diarrhea and malformations requiring surgical management; (iii) major chromosomal abnormalities (e.g., trisomy 13, 18 and 21); and (iv) other major congenital malformations (7, 12). Exclusion criteria comprised loss of central venous access for more than 72 consecutive hours that prevented continuation of parenteral nutrition.

### 2.3 Study design

This was a single-center retrospective observational cohort study comparing two sequential cohorts of newborns following a change in parenteral nutrition practice, following a study design previously adopted in our unit (13, 14). In November 2022, the NICU transitioned from IPN, compounded by the hospital pharmacy, to a commercially available SPN formulation (Numeta^®^ G13%E, Baxter B.V., Utrecht, The Netherlands). This transition was driven by institutional logistic and organizational considerations and did not involve any other modifications to the unit’s clinical protocols. Cohort A received IPN between January 2015 and October 2022. Cohort B received SPN between November 2022 and December 2024.

### 2.4 Nutritional protocol

In both groups, PN was initiated within 24 h after NICU admission. Prescriptions were adjusted daily by the attending neonatologists based on each infant’s clinical condition, laboratory findings, and body weight, using dedicated prescription software. Total macro- and micronutrient requirements via PN were calculated by subtracting the EN intake from the estimated total energy requirement. Parenteral nutrition was administered through central venous access. Total fluid intake was initiated at 70–90 mL/kg/day and increased by 10–20 mL/kg/day until reaching 150–180 mL/kg/day. In the IPN group, amino acids were started at 2.0 to 3.5 g/kg/day according to birth weight and advanced to a target of 3.5 to 4.0 g/kg/day, following the protocol previously described by our group (3). Electrolytes were prescribed in accordance with ESPGHAN recommendations and adjusted daily on the basis of serum values (15), with potassium withheld during the first 24 to 48 h of life.

The enteral feeding protocol was identical in both groups. Minimal enteral feeding (MEF) was initiated within 48 h after birth at 10–20 mL/kg/day and advanced by 20–30 mL/kg/day if tolerated. Fresh, unfortified human milk from the infant’s own mother was administered as soon as available; preterm formula was used when human milk was unavailable or insufficient. Human milk fortification was started once enteral intake reached 80 to 100 mL/kg/day, identically in both groups. Vitamin D was supplemented in all infants according to current recommendations, initially by the parenteral and subsequently by the enteral route, with an identical protocol in both groups (16).

### 2.5 Data collection

Prenatal, perinatal, and postnatal data were prospectively recorded using a standardized data collection form. Data on PN and EN were collected daily, including cumulative daily parenteral and enteral nutrient intake.

Specifically, the following data were extracted from the prospectively maintained NICU database: (i) prenatal and perinatal variables: maternal age, hypertensive disorders of pregnancy, gestational diabetes, maternal infectious risk, antibiotic prophylaxis, antenatal steroid administration, placental abruption, abnormal Doppler findings, intrauterine growth restriction, multiple pregnancy, mode of delivery, gestational age, birth weight, sex, Apgar score, cord pH, cord base excess, and respiratory distress syndrome requiring surfactant administration; (ii) daily nutritional intakes (day 0–7): parenteral and total fluid volume, sodium, potassium, calcium and phosphorus delivery, calcium-to-phosphorus ratio, cumulative parenteral protein intake, and proportion of enteral intake from own mother’s milk versus preterm formula; (iii) daily laboratory parameters during the first week of life: serum sodium, potassium, ionized calcium, phosphate, and acid–base status; (iv) postnatal co-treatments: blood transfusions and caffeine therapy. Ionized calcium was obtained from blood gas samples drawn as part of routine clinical monitoring; total calcium was not assessed in order to avoid additional blood sampling. Daily body weight during the first week of life was also recorded.

To evaluate PN-related complications, we collected data on the occurrence of hyponatremia (serum sodium <135 mmol/L), hyperkalemia (serum potassium >6 mmol/L), hypocalcemia (ionized calcium <1.1 mmol/L), and hyperphosphatemia (serum phosphate >3.1 mmol/L) (1, 15, 17–20). Data on survival and prematurity-related morbidities were also collected. Overall morbidity was defined as the presence of at least one major complication of prematurity, including necrotizing enterocolitis (NEC, Bell stage ≥2), intraventricular hemorrhage (IVH, grade ≥2), periventricular leukomalacia (PVL), culture-proven sepsis, retinopathy of prematurity (ROP, stage ≥3), or bronchopulmonary dysplasia (BPD) (21–23). Diagnoses were established according to standard criteria by clinicians blinded to the study design and objectives.

### 2.6 Statistical analysis

Statistical analyses were performed using SPSS for Windows, version 27.0 (IBM Corp., Chicago, IL, USA). Normality was assessed using the Shapiro–Wilk test. Continuous variables were summarized as mean ± SD or median (IQR) and compared using Student’s *t*-test, Mann–Whitney *U* test, or Wilcoxon test as appropriate. Categorical variables were compared using the chi-square test.

Four separate binary logistic regression models (Enter method) were built in SPSS to identify independent risk factors for hyponatremia, hyperkalemia, hypocalcemia and hyperphosphatemia. For each outcome a specific set of clinically relevant covariates was entered into the model, as detailed in Figure 1; the corresponding events-per-variable ratios were 28, 15, 15 and 20, respectively, all above the conventional threshold of 10. Because the hyponatremia model was affected by quasi-complete separation (31/33 SPN neonates with hyponatremia), it was additionally re-estimated using Firth-penalized logistic regression as a sensitivity analysis (Supplementary Table S1). All statistical tests were two-sided; *p* < 0.05 was considered significant. Analyses were performed on complete cases. The statistician was blinded to the study objectives and patient identifiers were disclosed only after completion of the statistical analysis. A prespecified interim analysis was planned after 12 months from the change in clinical practice in order to assess the safety profile of the new PN strategy. A *post-hoc* power analysis was performed for each of the four primary outcomes (*z*-test for the difference between two independent proportions, two-sided *α* = 0.05, *n*_1_ = 33, *n*_2_ = 362). The achieved power was >99.9% for hyponatremia, 90.1% for hypocalcemia, 82.1% for hyperphosphatemia and 55.7% for hyperkalemia, corresponding to observed effect sizes (Cohen’s *h*) of 1.15, 0.59, 0.52 and 0.38, respectively. With this sample-size configuration the study could detect at 80% power a minimum effect size of *h* ≈ 0.51, equivalent to approximately a 24-percentage-point difference in event rate.

**Figure 1:**
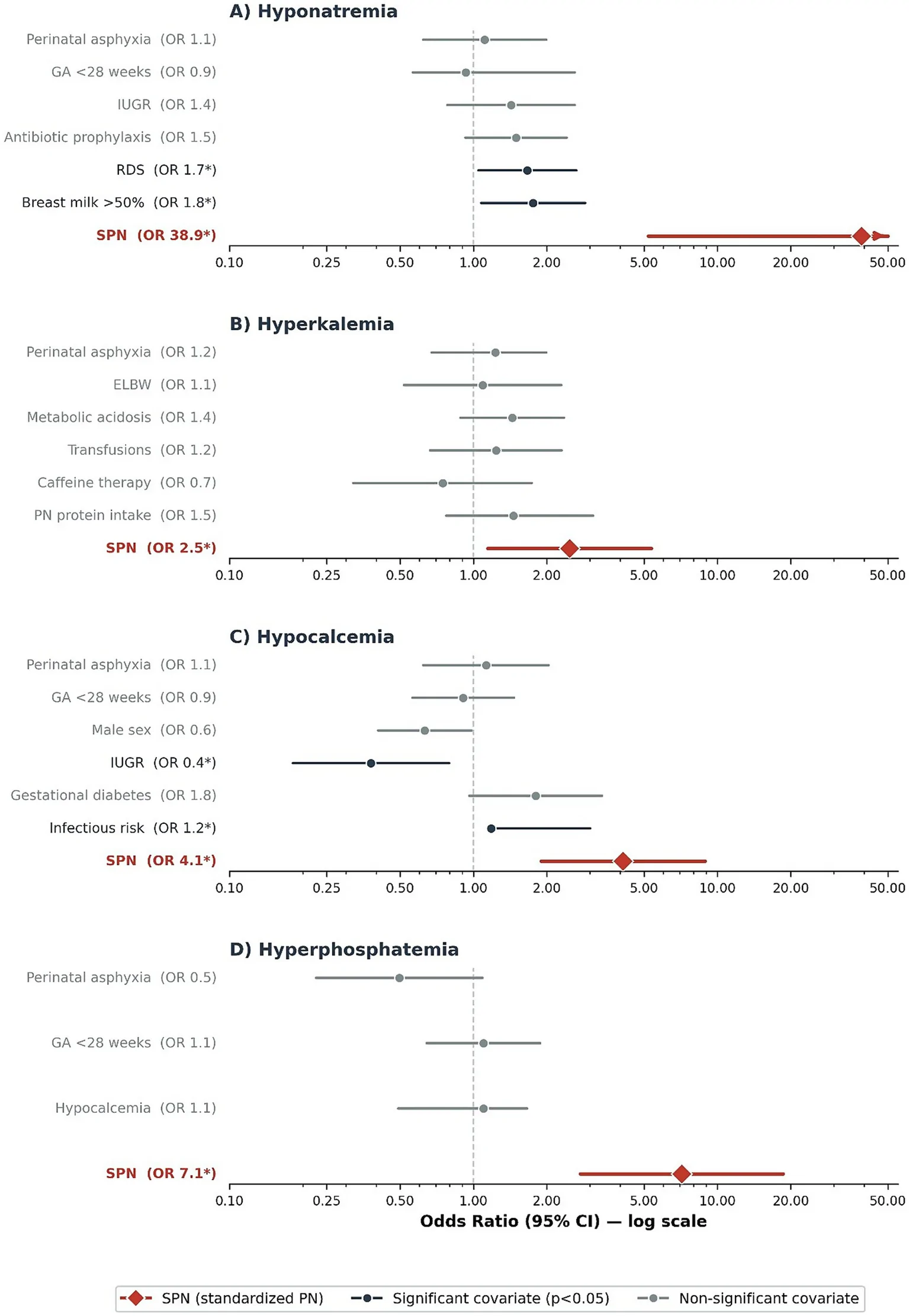
Forest plot of binary logistic regression analyses for **(A)** hyponatremia, **(B)** hyperkalemia, **(C)** hypocalcemia, and **(D)** hyperphosphatemia. Odds ratios with 95% confidence intervals are displayed on a logarithmic scale. SPN (red diamonds) was independently associated with all four outcomes. Significant covariates (*p* < 0.05) are shown in dark blue; non-significant covariates in grey. The dashed vertical line indicates OR = 1. *p < 0.05.

## 3 Results

After 12 months from the change in clinical practice, the prespecified interim analysis was performed. The results are presented below and, in light of the safety concerns observed in the SPN group, led to discontinuation of the practice change.

### 3.1 Baseline characteristics

Baseline prenatal and postnatal clinical characteristics of the IPN and SPN groups, including known perinatal risk factors for adverse neonatal outcomes are summarized in Table 1. No statistically significant differences were observed between the two groups for any of the variables assessed.

**Table 1:** Baseline clinical characteristics of the study population.

Characteristics	IPN (*n* = 362)	SPN (*n* = 33)	*p*
Maternal age ≥35 years, *n* (%)	167 (46.1)	11 (33.3)	0.103
Placental abruption, *n* (%)	33 (9.1)	3 (9.1)	1.000
Abnormal Doppler findings, *n* (%)	63 (17.4)	6 (18.2)	0.815
Maternal infectious risk, *n* (%)	129 (35.6)	10 (30.3)	0.575
Antibiotic prophylaxis, *n* (%)	108 (29.8)	7 (21.2)	0.423
Hypertensive disorders, *n* (%)	82 (22.7)	8 (24.2)	0.830
Gestational diabetes, *n* (%)	40 (11.0)	6 (18.2)	0.252
IUGR, *n* (%)	56 (15.5)	6 (18.2)	0.623
Antenatal steroids, *n* (%)	238 (65.7)	17 (51.5)	0.128
Cesarean section, *n* (%)	315 (87.0)	26 (78.8)	0.414
Multiple pregnancy, *n* (%)	118 (32.6)	10 (30.3)	0.848
Cord pH < 7.2, *n* (%)	62 (17.1)	7 (21.2)	0.962
Cord base excess	−5.8 (−6.3 to −5.3)	−5.3 (−7.2 to −3.4)	0.537
APGAR at 5′ < 5, *n* (%)	28 (7.7)	6 (18.1)	0.321
Male sex, *n* (%)	198 (54.7)	17 (51.5)	0.855
GA < 28 weeks, *n* (%)	308 (85.1)	25 (75.8)	0.710
ELBW, *n* (%)	101 (27.9)	12 (36.4)	0.202
Birth weight, grams	1222.0 (1184.4–1259.6)	1179.3 (1036.7–1321.9)	0.523

IPN, individualized parenteral nutrition; SPN, standardized parenteral nutrition; IUGR, intrauterine growth restriction; ELBW, extremely low birth weight (≤1,000 g); GA, gestational age. Data are presented as *n* (%) or mean (95% CI). *p < 0.05.

### 3.2 Nutritional intakes

The main nutritional characteristics of the study population during the first week of life are reported in Table 2. The SPN group received a significantly lower volume of parenteral nutrition compared with the IPN group, whereas no significant differences were observed in total volume (PN + EN). Weight at day 7 did not differ between groups (1,148 ± 351 vs. 1,170 ± 354 g; *p* = 0.755). During the first week of life, the SPN group received significantly lower amounts of sodium and calcium. The calcium-to-phosphorus ratio was also significantly different between groups. Cumulative parenteral protein delivery during the first week was also significantly lower in the SPN group (mean 9.68 vs. 13.96 g/kg/0–7 days; *p* = 0.012). The cumulative volume of mother’s own milk delivered during the first week of life was comparable between the two groups (IPN: median 10.9 mL/kg, IQR 0.0–63.8; SPN: median 5.0 mL/kg, IQR 0.0–50.0; *p* = 0.400). Mean daily parenteral micronutrient intakes during the first week of life are shown in Figure 2. The IPN group received consistently higher intakes of sodium, calcium, and phosphorus from day 0 through day 7. Of note, potassium intake in the IPN group was intentionally withheld during the first 24–48 h of life (near-zero on days 0–1), whereas SPN delivered potassium from the first administration due to its fixed composition (Figure 2B). From day 3 onward, IPN potassium intake exceeded that of SPN.

**Table 2:** Parenteral micronutrient intakes during the first week of life.

Nutritional variables	IPN (*n* = 362)	SPN (*n* = 33)	*p*
PN volume, mL/kg/0–7 days	579.6 (557.3–601.8)	404.8 (350.0–459.7)	<0.001*
Total volume, mL/kg/0–7 days	579.6 (557.3–601.8)	586.7 (484.2–689.1)	0.862
Sodium, mmol/kg/0–7 days	13.29 (12.3–14.2)	6.7 (5.2–8.2)	<0.001*
Potassium, mmol/kg/0–7 days	7.7 (7.1–8.2)	6.4 (4.9–7.8)	0.178
Calcium, mg/kg/0–7 days	235.3 (216.3–254.2)	153.7 (120.1–187.4)	0.012*
Phosphorus, mg/kg/0–7 days	290.1 (214.1–366.1)	115.8 (85.9–145.8)	0.188
Calcium/phosphorus ratio, 0–7 days	0.81 (0.81–0.91)	1.33 (1.27–1.38)	<0.001*

IPN, individualized parenteral nutrition; SPN, standardized parenteral nutrition; PN, parenteral nutrition. Data are presented as mean (95% CI). *p < 0.05.

**Figure 2:**
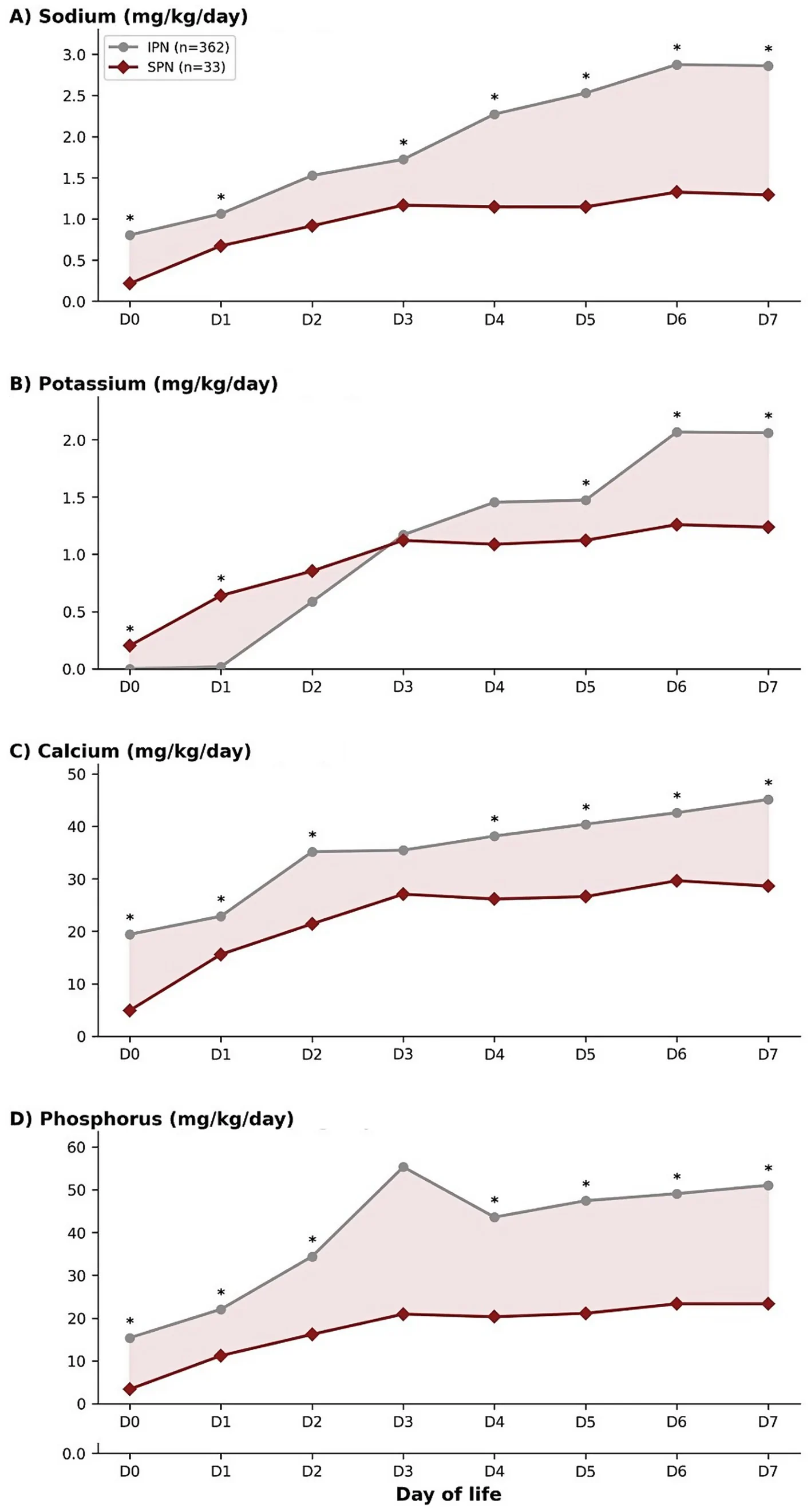
Mean daily parenteral micronutrient intakes [**(A)** sodium; **(B)** potassium; **(C)** calcium; **(D)** phosphorus] during the first week of life in preterm neonates receiving individualized (IPN, *n* = 362) versus standardized (SPN, *n* = 33) parenteral nutrition. The shaded area represents the gap between formulations. *p < 0.05.

### 3.3 Electrolyte disturbances

The incidence of electrolyte disturbances is shown in Figure 3. The SPN group showed a significantly higher incidence of all electrolyte imbalances compared with the IPN group: hyponatremia (93.9% vs. 46.1%, *p* < 0.01), hyperkalemia (42.4% vs. 24.5%, *p* = 0.019), hypocalcemia (51.5% vs. 23.4%, *p* < 0.001), and hyperphosphatemia (42.4% vs. 18.7%, *p* < 0.01).

**Figure 3:**
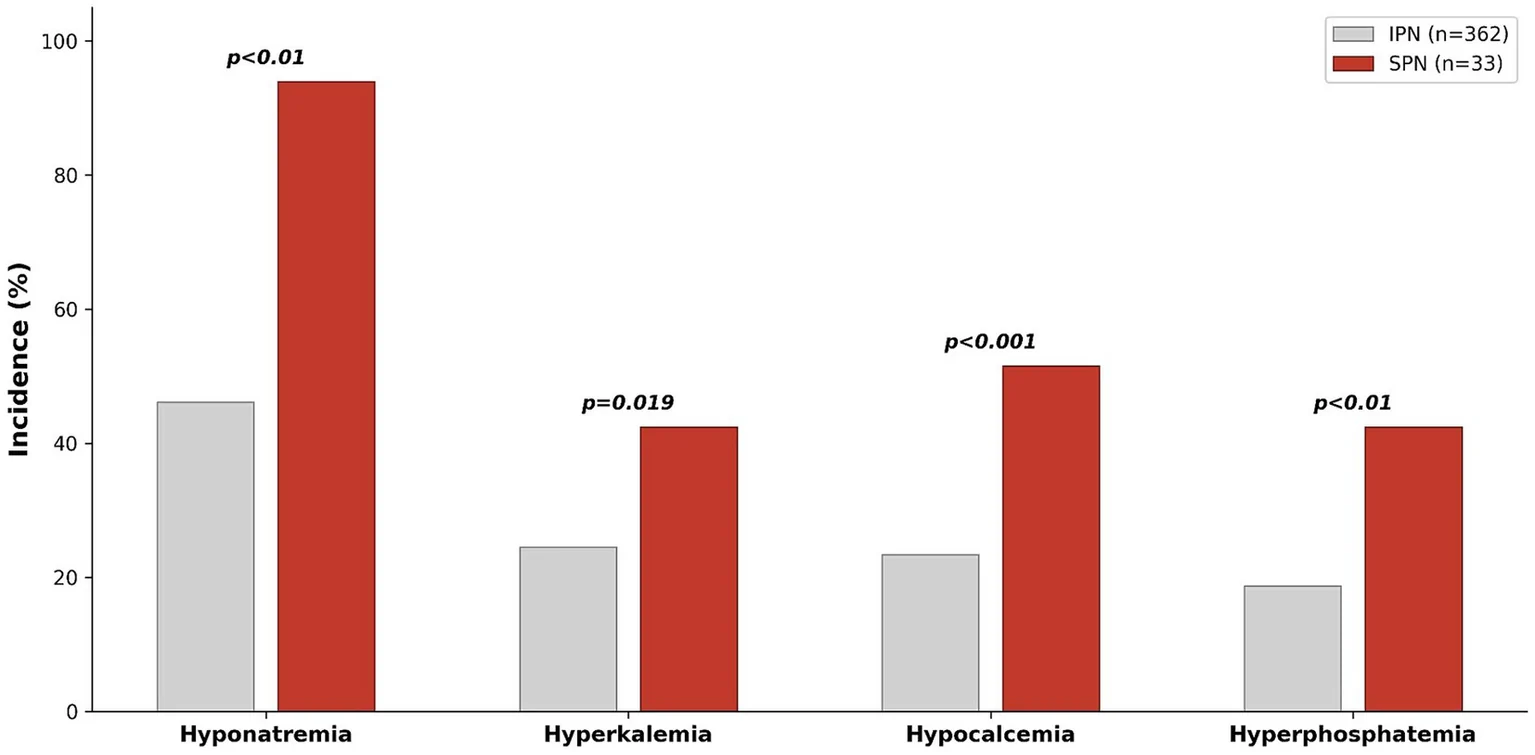
Incidence of electrolyte disturbances in preterm neonates receiving IPN (*n* = 362) versus SPN (*n* = 33) during the first week of life. Hyponatremia: serum sodium <135 mmol/L; hyperkalemia: serum potassium >6 mmol/L; hypocalcemia: ionized calcium <1.1 mmol/L; hyperphosphatemia: serum phosphate >3.1 mmol/L. *p*-values from chi-square test.

### 3.4 Multivariate analysis

The binary logistic regression analyses are reported in Figures 1A–D. In all four models, SPN remained independently associated with the development of electrolyte disturbances after adjustment for the main confounding factors.

For hyponatremia (Figure 1A), SPN was the strongest independent risk factor (OR 38.9; 95% CI: 5.21–290.7; *p* < 0.001). Respiratory distress syndrome and breast milk intake >50% also emerged as significant predictors.

For hyperkalemia (Figure 1B), SPN was the only independently associated factor (OR 2.48; 95% CI: 1.14–5.36; *p* = 0.021). For hypocalcemia (Figure 1C), in addition to SPN (OR 4.11; 95% CI: 1.89–8.92; *p* < 0.001), IUGR and maternal infectious risk were independently associated. For hyperphosphatemia (Figure 1D), SPN was the only independently associated factor (OR 7.14; 95% CI: 2.74–18.57; *p* < 0.001).

## 4 Discussion

SPN was associated with a higher rate of metabolic complications and with lower protein intake than IPN. In light of these safety findings, the interim analysis prompted discontinuation of the practice change in favor of SPN and restoration of the previous IPN-based approach.

The analytic cohort was substantially unbalanced, reflecting the early discontinuation of the SPN arm prompted by the prespecified interim safety analysis. This imbalance should be considered when interpreting the multivariable estimates, particularly the markedly elevated odds ratio for hyponatremia. Its wide confidence interval reflects the limited size of the SPN cohort, not the direction of the association.

The present study is, to our knowledge, the first to specifically investigate electrolyte and mineral disturbances as the primary endpoint in a direct comparison between SPN and IPN in preterm neonates, employing multivariate analysis to identify independent risk factors for each specific electrolyte abnormality. The existing literature is limited to non-randomized, single-center observational studies that primarily evaluated macronutrient intake and growth outcomes, with electrolyte balance addressed only as a secondary or ancillary endpoint (10, 11). This gap is clinically significant, as electrolyte disturbances in the neonatal period have been associated with acute neurological injury, oxidative stress, and long-term neurodevelopmental consequences, including seizure disorders and headache in childhood (24–26).

Smolkin et al. (27) compared 140 VLBW neonates receiving IPN versus five different SPN formulations and reported that IPN was associated with better growth, shorter duration of exclusive PN, and fewer electrolyte corrections. However, electrolyte stability was assessed as a secondary outcome without multivariate adjustment.

The only previous study explicitly aimed at comparing fluid and electrolyte balance between SPN and IPN was conducted by Iacobelli et al. (28) in 107 preterm infants less than 33 weeks of gestation. The authors found a higher incidence of non-oliguric hyperkalemia in the IPN group, which they attributed to lower early amino acid and energy intakes in IPN. The SPN formulations used in that study provided higher amino acid, energy and sodium contents than IPN, a compositional pattern opposite to that observed with Numeta^®^ G13%E in the present investigation. This compositional difference may explain the contrasting direction of findings. No multivariate models were used to control for potential confounding factors (28).

Yeung et al. (29) reported no significant differences in biochemical laboratory parameters between SPN and IPN groups, despite lower sodium and higher calcium and phosphate intakes in the SPN group from day 4 onward. This is in line with our finding of significantly lower sodium intake in the Numeta^®^ G13%E group. However, because Yeung et al. (29) did not specifically assess individual electrolyte disturbances as clinical outcomes, subtle yet clinically meaningful differences in the incidence of hyponatremia, hyperkalemia, hypocalcemia, or hyperphosphatemia may have been missed.

Evering et al. (30) conducted the largest comparison to date (299 preterm infants <32 weeks), including a group receiving Numeta^®^ G13%E, and found no clinically relevant differences in electrolyte concentrations between IPN and SPN. However, growth was the primary endpoint and electrolyte concentrations were explicitly designated as secondary endpoints. The discrepancy with our findings may also be related to the timing of biochemical assessment: in Evering et al. (30), electrolyte values were mainly evaluated during the first three postnatal days, whereas our analysis considered the entire first week of life.

Additionally, Senterre et al. (20) conducted a single-arm prospective study in 102 VLBW infants using a specifically designed, optimized SPN solution, not a commercially available product, and demonstrated that even under these favorable conditions, improvement in electrolyte homeostasis required deliberate upward adjustment of electrolyte supply. If a purpose-built SPN still required supplementation to maintain homeostasis, the limitations of a fixed-composition commercial product, in which individual electrolyte components cannot be modified, are clearer still. The findings of Senterre et al. (20) are consistent with our own and suggest that the electrolyte composition of commercially available SPN formulations may be inadequate for the metabolic demands of preterm neonates during the first week of life.

### 4.1 Hyponatremia

Several studies suggest that the sodium content of standardized parenteral solutions may be insufficient during the first week of life (15, 17). This phenomenon may be partially explained by early protein intake, which induces a positive nitrogen balance with intracellular sodium accumulation of 1.0–1.5 mmol/kg/day (17). The markedly elevated odds ratio for SPN in our analysis (OR 38.9) likely reflects the substantially lower cumulative sodium delivery documented in Table 2, combined with the inability to adjust sodium supplementation independently within the fixed Numeta^®^ G13%E formulation.

Respiratory distress syndrome emerged as an additional independent risk factor for hyponatremia (OR 1.67), likely through arginine vasopressin (AVP) secretion triggered by hypovolemia and hypoxia, ultimately resulting in dilutional hyponatremia. Enteral feeding with human milk >50% of total intake was also independently associated with hyponatremia (OR 1.76), as human milk contains suboptimal sodium concentrations for preterm and VLBW infants, who exhibit para-physiological increased renal tubular sodium losses. In the SPN group human milk represented a proportionally larger share of a smaller parenteral electrolyte load, amplifying the relative contribution of the enteral sodium deficit. Preterm infants receiving a fixed-composition SPN together with a substantial proportion of human milk therefore have increased sodium requirements, and early sodium supplementation should be considered in this setting.

### 4.2 Hyperkalemia

Regarding hyperkalemia, SPN was the only statistically significant risk factor (OR 2.48). Potassium levels are known to be influenced by protein intake during the first days of life (17). In the IPN group, potassium was intentionally withheld during the first 24–48 h of life, a practice aligned with standard neonatal care that defers potassium supplementation until adequate urine output is established. In contrast, SPN, being a fixed-composition formulation, delivers potassium from the first administration, as the clinician cannot selectively remove individual electrolytes.

The electrolyte disturbances observed in the SPN group may also be attributable to suboptimal protein intake. As previously described in the context of the PI-Feeding syndrome, protein deficiency promotes cellular protein degradation with consequent release of intracellular potassium into the extracellular space (17). Consistent with this hypothesis, our cohort received a significantly lower parenteral protein intake in the SPN group during the first week of life (Section 3.2). However, since a formal mediation analysis was not performed, this interpretation should be regarded as hypothesis-generating and based on prior evidence rather than directly demonstrated by the present dataset.

### 4.3 Hypocalcemia

With respect to hypocalcemia, SPN was the strongest independent risk factor (OR 4.11). The cessation of placental calcium transfer leads to a decrease in serum calcium levels, reaching a nadir 24–30 h after birth in preterm infants (18). In term neonates, this physiological decline triggers a compensatory increase in parathyroid hormone (PTH) levels (19). In contrast, preterm infants do not exhibit a comparable PTH response, and renal immaturity results in increased urinary calcium excretion. When combined with insufficient calcium intake due to inadequate PN as documented by the significantly lower calcium delivery in the SPN group (Table 2); this mechanism may contribute to early hypocalcemia. Ionized calcium exists in equilibrium with the protein-bound fraction, and alkalosis increases calcium binding to albumin whereas acidosis has the opposite effect. In our cohorts acid–base status at birth was comparable between groups and perinatal asphyxia was included as a covariate in the multivariable model, making a differential contribution of this mechanism unlikely.

Intrauterine growth restriction was also independently associated with hypocalcemia (OR 0.38), likely through impaired placental calcium transfer during fetal life. Maternal infectious risk emerged as an additional significant predictor (OR 1.18), contributing through inflammatory mechanisms that interfere with calcium homeostasis.

### 4.4 Hyperphosphatemia

Regarding hyperphosphatemia, SPN was the only independently associated factor (OR 7.14), after adjustment for confounders including perinatal asphyxia, gestational age, and serum calcium levels (11). Notably, no significant differences in phosphorus intake were observed between the two groups (Table 2), suggesting that the pathogenesis of hyperphosphatemia in the SPN group cannot be attributed to excessive exogenous phosphorus delivery. Rather, it may reflect insufficient protein intake resulting in persistent cellular catabolism, with consequent phosphate efflux into the extracellular compartment (17). The higher Ca:P ratio in SPN, which coexists with both hypocalcemia and hyperphosphatemia, is consistent with an endogenous mechanism: an absolute calcium deficit combined with a phosphorus excess of endogenous, not exogenous, origin. The Ca:P ratio describes the proportion between the two minerals but not the adequacy of their absolute supply. In the SPN group both calcium and phosphorus intakes were lower than in the IPN group, and the higher ratio reflects a proportionally greater reduction in phosphorus than in calcium rather than an optimised mineral supply. With a fixed-composition formulation the absolute deficit cannot be corrected without simultaneously altering the ratio. This mechanistic interpretation, although supported in our cohort by the significantly lower parenteral protein intake documented in the SPN group (Section 3.2), remains hypothesis-generating, as a formal mediation analysis was not performed in the present study.

### 4.5 Strengths and limitations

The present study should be regarded as hypothesis-generating rather than confirmatory. The observational, before-and-after design, based on two sequential cohorts spanning different time periods, does not allow causal inference between the type of parenteral nutrition and the development of electrolyte and mineral disturbances. Although no other modifications were made to the NICU clinical protocols during the study period, secular trends in neonatal care and unmeasured changes in clinical thresholds cannot be fully excluded as sources of residual temporal confounding. The findings reported here should therefore be interpreted as associations rather than causal effects, and warrant confirmation in prospective, ideally randomized, multicenter studies.

The present study is the first to adopt electrolyte and mineral disturbances as the primary study endpoint in a direct comparison between SPN and IPN in preterm neonates. Unlike previous studies, our design employed binary logistic regression to identify independent risk factors for each specific electrolyte disturbance after adjustment for relevant clinical confounders. Additionally, the day-by-day analysis of micronutrient intakes during the first week of life provides a temporal resolution rarely reported in previous comparative studies.

Several limitations should be acknowledged. This is a single-center retrospective study with a non-randomized design and sequential cohorts, which limits causal inference. The considerable disparity in sample size between the IPN (*n* = 362) and SPN (*n* = 33) groups may affect statistical power; however, the consistency of the findings across all regression models supports their robustness. The *post-hoc* power analysis (Section 2.6) confirmed adequate power for hyponatremia, hypocalcemia and hyperphosphatemia, whereas the comparison for hyperkalemia was underpowered (55.7%); this specific association should therefore be regarded as hypothesis-generating. Effects smaller than *h* ≈ 0.51 could not be reliably detected. This imbalance reflects our prespecified interim safety analysis, which led to early discontinuation of the SPN arm once the unfavourable safety signal emerged, in line with the ethical principle of equipoise. As a result, the cohort imbalance affected the precision of the multivariable estimates, as shown by the wide confidence interval of the hyponatremia odds ratio, but not the direction of the associations. The SPN formulation used (Numeta^®^ G13%E) represents a specific commercially available product, and results may not be directly generalizable to other standardized solutions with different electrolyte compositions (28). Finally, the sequential cohort design introduces potential temporal confounding, although no changes in NICU protocols other than the PN formulation were implemented during the study period. A practical implication is that, when a fixed-composition SPN is used the need for additional supplementation of sodium, calcium and phosphate reduces the operational simplicity and safety advantages traditionally attributed to SPN, partially offsetting one of its main rationales (31). Total calcium was not measured, as ionized calcium was available from routine blood gas analysis and additional venipuncture was avoided. Serum magnesium was not systematically available and was therefore not included among the study outcomes; a contribution of magnesium status to the hypocalcemia observed in our cohorts cannot be excluded.

Despite these limitations, the present study provides exploratory but clinically meaningful data on a topic that is highly relevant, yet still insufficiently explored: the association between different parenteral nutrition strategies and electrolyte or mineral disturbances in preterm neonates. Our findings contribute preliminary evidence that can support the generation of new hypotheses and inform the design of future prospective, randomized, and multicenter studies on the electrolyte safety of fixed-composition SPN formulations in this vulnerable population.

## 5 Conclusion

This cohort study suggests that SPN was associated with a significantly higher risk of electrolyte and mineral imbalances, including hyponatremia, hyperkalemia, hypocalcemia, and hyperphosphatemia, compared with individualized formulations. Where individualized parenteral formulations are not available, clinicians should be aware of this risk and consider preventive strategies, including targeted electrolyte and mineral supplementation. These findings warrant confirmation through larger, prospective, multicenter studies specifically designed to evaluate the electrolyte safety of commercially available standardized parenteral nutrition formulations in preterm neonates.

## Data Availability

The raw data supporting the conclusions of this article will be made available by the authors, without undue reservation.
