# Supplementary material for: Effects of standardized parenteral nutrition on electrolyte balance: an observational study in preterm neonates
Source: Front Nutr. 2026 Oct 5;13:1849396. doi: 10.3389/fnut.2026.1849396 (PMC13677517; doi:10.3389/fnut.2026.1849396)
Supplement: Supplementary file 1 [file Table_1.DOCX]

**Supplementary Table S1.**

*Firth-penalized logistic regression sensitivity analysis for the hyponatremia model.*

| **Covariate** | **β** | **OR** | **95% CI** | **p** |
| --- | --- | --- | --- | --- |
| **Hyponatremia (IpoNaT1) — Firth penalized logistic regression \| n=380, events=196 (51.6%)** | | | | |
| (Intercept) | −0.660 | 0.517 | 0.348 – 0.768 | 0.001 |
| Perinatal asphyxia (cord pH <7.2) | 0.104 | 1.110 | 0.622 – 1.979 | 0.724 |
| Gestational age <28 weeks | −0.068 | 0.934 | 0.581 – 1.502 | 0.778 |
| IUGR | 0.351 | 1.420 | 0.783 – 2.576 | 0.248 |
| Antibiotic prophylaxis | 0.403 | 1.496 | 0.930 – 2.406 | 0.097 |
| RDS (endotracheal surfactant) | 0.508 | 1.662 | 1.053 – 2.623 | 0.029* |
| Human milk intake >50% (week 1) | 0.562 | 1.755 | 1.072 – 2.872 | 0.025* |
| **SPN (Numeta® G13%E)** | **3.241** | **25.57** | **4.82 – 135.70** | **0.0001*** |

*Abbreviations: SPN, standardized parenteral nutrition; IUGR, intrauterine growth restriction; RDS, respiratory distress syndrome; OR, odds ratio; CI, confidence interval. *p<0.05.*
